# Biomarkers of thyroid function and autoimmunity for predicting high-risk groups of thyroid cancer: a nested case–control study

**DOI:** 10.1186/1471-2407-14-873

**Published:** 2014-11-24

**Authors:** Young Ae Cho, Sun-Young Kong, Aesun Shin, Jeonghee Lee, Eun Kyung Lee, You Jin Lee, Jeongseon Kim

**Affiliations:** Division of Cancer Epidemiology and Prevention, Molecular Epidemiology Branch, Research Institute, National Cancer Center, 323 Ilsan-ro, Ilsandong-gu, Goyang-si 410-769, Gyeonggi-do, Korea; Division of Cancer Epidemiology and Prevention, Translational Epidemiology Branch, Research Institute, National Cancer Center, Goyang-si 410-769, Gyeonggi-do, Korea; Department of Laboratory Medicine, Center for Diagnostic Oncology, Hospital, National Cancer Center, Goyang-si 410-769, Gyeonggi-do, Korea; Department of Preventive Medicine, College of Medicine, Seoul National University, Seoul, 110-799 Korea; Center for Thyroid Cancer, National Cancer Center, Goyang-si 410-769, Gyeonggi-do, Korea

**Keywords:** Thyroid cancer, Biomarkers, Thyroid function, Autoimmunity, TPOAb

## Abstract

**Background:**

A remarkable increase in the number of thyroid cancer cases has been reported in recent years; however, the markers to predict high-risk groups have not been fully established.

**Methods:**

We conducted a case–control study (257 cases and 257 controls) that was nested in the Cancer Screenee Cohort Study between August 2002 and December 2010; the mean follow-up time for this study was 3.1 ± 2.2 years. The levels of total triiodothyronine (TT3), free thyroxine (FT4), thyroid-stimulating hormone (TSH), thyroglobulin (Tg), anti-thyroperoxidase antibody (TPOAb), and anti-thyroglobulin antibody (TgAb) were measured using samples with pre-diagnostic status. Logistic regression models were used to examine the association between thyroid function/autoimmunity and thyroid cancer risk.

**Results:**

When the markers were categorized by the tertile distributions of the control group, the highest tertile of FT4 (OR = 1.73, 95% CI = 1.11 - 2.69) and the middle tertile of TSH (OR = 1.77, 95% CI = 1.14 - 2.74) were associated with an increased risk of thyroid cancer by multivariate analyses. In addition, an elevated risk for thyroid cancer was found in subjects with TPOAb levels above 30 IU/mL (OR = 8.47, 95% CI = 5.39 - 13.33 for 30–60 IU/mL and OR = 4.48, 95% CI = 2.59 - 7.76 for ≥60 IU/mL). Stratified analyses indicated that some of these associations differed by sex, BMI, smoking status, and the duration of follow-up.

**Conclusions:**

This study demonstrated that the levels of biomarkers of thyroid function/autoimmunity, particularly the presence of TPOAb, might be used as diagnostic markers for predicting thyroid cancer risk. Our findings suggest that careful monitoring of thyroid biomarkers may be helpful for identifying Korean populations at high-risk for thyroid cancer.

**Electronic supplementary material:**

The online version of this article (doi:10.1186/1471-2407-14-873) contains supplementary material, which is available to authorized users.

## Background

Thyroid cancer is the most frequent cancer among endocrine tumors, and its incidence has been greatly increasing in many countries [1]. In particular, the incidence of thyroid cancer in Korea has increased rapidly and has become one of the highest in the world [2]. Although the increased incidence rate of thyroid cancer is partly attributed to the increased detection of subclinical cancer resulting from advanced diagnostic technologies [3], studies have reported a true increase in thyroid cancer incidence due to changes in lifestyle or environmental factors (e.g., iodine intake, exposure to radiation) [4, 5].

Recently, an effort has been made to predict the risk of thyroid cancer using the markers of thyroid function/autoimmunity [6–9]. Although the findings were inconsistent, several studies found biomarkers that predicted thyroid cancer. Some studies have reported that higher levels of thyroid-stimulating hormone (TSH) are associated with an increased risk of thyroid malignancy [6, 7], possibly because of its role in affecting thyroid cell differentiation and proliferation or in stimulating angiogenesis [10]. Other studies have suggested that thyroid autoantibodies could be used as predictors of thyroid cancer risk based on the association between thyroid autoimmune disease and thyroid cancer [9]. However, most studies have investigated these associations retrospectively, which has the potential for selection and referral biases.

In this study, we aimed to investigate whether blood markers representing thyroid function and autoimmunity could predict thyroid malignancy. We designed a nested case–control study, which was affected little by bias, to validate blood markers for thyroid malignancy.

## Methods

### Study population

We conducted a nested case–control study on participants in the ongoing Cancer Screenee Cohort Study (CSCS) between August 2002 and December 2010, which had a mean time of follow-up of 3.1 ± 2.2 years. The CSCS is a prospective cohort study consisting of participants of the Cancer Screening Program at the National Cancer Center in South Korea. Participants were aged 30 years or older, underwent health-screening examinations, and were screened for selected cancers. All of the participants were asked to complete a self-administered questionnaire at the baseline evaluation. The data collected in the baseline evaluation included socio-demographic characteristics, personal and family medical history, lifestyle factors, and reproductive factors. A total of 22,085 subjects provided written informed consent and provided a blood sample for study participation.

### Ascertainment of cases and selection of controls

**Figure 1:**
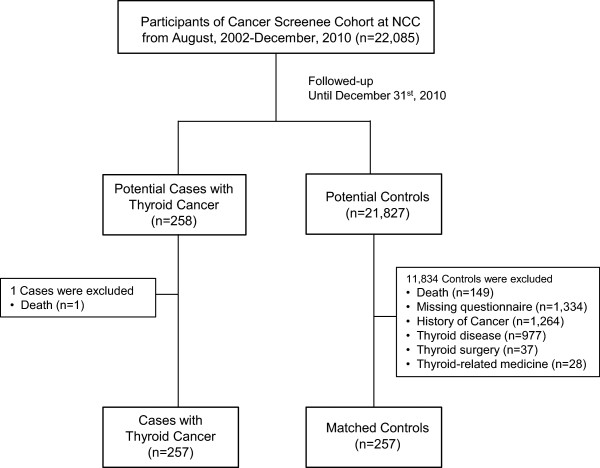
**Flowchart of the sampling process of the nested case-control samples.**

Potential cases diagnosed with thyroid cancer (ICD10 code C73) were ascertained by linkage to the Korea Central Cancer Registry (KCCR) database, which was used to identify the incidence of cancer in Korea. Among 258 thyroid cancer patients, 257 patients were selected after excluding those who were dead. Among the potential controls (n = 21,827) who were not diagnosed with thyroid cancer, 3,740 participants were excluded because of the following reasons: death, missing questionnaire data, history of other cancers, any thyroid disease, thyroid surgery, or thyroid-related medicine. For each case, one control among the remaining 18,807 participants who was matched by entry age (same age) and sex was selected. In total, 257 incident cases and 257 controls were used for the final biomarker analysis (Figure 1). The participants were followed up from the date of blood collection until December 31, 2010. The study procedure was approved by the institutional review board of the National Cancer Center (NCCNCS 13–698).

### Laboratory procedures

Blood samples were collected at the baseline evaluation and stored at -80°C until analysis. The serum concentrations of the following six biomarkers were measured for both cases and controls: total triiodothyronine (TT3), thyroid-stimulating hormone (TSH), free thyroxine (FT4), thyroglobulin (Tg), anti-thyroglobulin antibody (TgAb), and anti-thyroperoxidase antibody (TPOAb). We selected these biomarkers of thyroid function/autoimmunity based on their associations with thyroid cancer that had been reported in previous studies [6–9, 11].

The serum concentrations of TT3, TSH, Tg, FT4, TgAb, and TPOAb were measured using an electrochemiluminescence immunoassay (ELCLIA; Molecular Analytics E170, Roche kit, Roche, Mannheim, Germany), which had reference (normal) ranges of 0.82 - 2.0 ng/mL for TT3, 0.27 - 4.20 μIU/mL for TSH, 0.93 - 1.70 ng/dL for FT4, and 1.4 - 78.0 ng/mL for Tg. TgAb was defined as negative if ≤115.0 IU/mL, and TPOAb was defined as negative if ≤34.0 IU/mL. The detection limits were 20 IU/mL for TgAb and 30 IU/mL for TPOAb.

### Statistical methods

The general characteristics of the study participants and the risk factors for thyroid cancer were compared using t-tests for continuous variables and chi-square tests for categorical variables. To evaluate the association between serum biomarkers and thyroid cancer risk, serum levels of TT3, FT4, TSH, and Tg were categorized into three groups based on those of the control group. The antibody titers for TgAb and TPOAb were also categorized into tertiles: the lowest tertile (under detection limit; 20 IU/mL for TgAb and 30 IU/mL for TPOAb), the middle tertile (over detection limit - < 60 IU/mL), and the highest tertile (>60 IU/mL). Then, we performed unconditional and conditional logistic regressions and calculated odds ratios (ORs) and 95% confidence intervals (CIs) using univariate and multivariate analyses. The lowest levels of each biomarker were used as references. The multivariate unconditional logistic regression models were adjusted for age, sex, body mass index (BMI) (<23, 23 - < 25, and ≥25 kg/m^2^), and cigarette smoking (nonsmoker, former smoker, and current smoker). To analyze the association between Tg levels and cancer risk, we excluded subjects who were positive for TgAb because the presence of TgAb hampers the usefulness of serum Tg as a tumor marker [12]. To explore potential modifying factors, analyses stratified according to sex, BMI (<23 and ≥23 kg/m^2^), and smoking status (nonsmoker and former/current smoker) were conducted; these factors showed different distributions between cases and controls in this study and have been reported to affect thyroid cancer risk [13, 14]. We also conducted an analysis stratified by the duration of follow-up. To examine the role of TPOAb in the association between thyroid cancer risk and other biomarkers, we also conducted analyses stratified by the presence of TPOAb. Because unconditional regression produced more stable results for the different subanalyses [15], only the results from the unconditional analyses are presented in the tables. We verified that both the conditional and unconditional approaches gave approximately the same results for the entire dataset.

All statistical analyses were performed using SAS 9.1 software (SAS Institute Inc., Cary, NC). A two-sided *P*-value of less than 0.05 was regarded as statistically significant.

## Results

This study included 257 cases and 257 controls, of whom 70% were women and 30% were men. We examined the differences in the general characteristics of the study subjects according to thyroid cancer status (Table 1). The mean age for cases and controls was 49.4 ± 8.9 years. The cases were more likely to have a higher BMI than the controls (*P* = 0.019); however, no differences with respect to other variables were observed between the cases and controls.

**Table 1:** **General characteristics of the study subjects**

	Controls (n = 257)	Cases (n = 257)	***P*** -value
Age (years), means	49.4 ± 8.9	49.4 ± 8.9	0.994
BMI (kg/m^2^)			
<23	125(48.6)	95(37.0)	0.019
23 - <25	68(26.5)	75(29.2)	
≥ 25	64(24.9)	87(33.9)	
Family history of cancer (yes)^a^	93(41.0)	108(46.4)	0.245
Family history of thyroid cancer (yes)^a^	5(2.2)	6(2.6)	0.794
Educational level			
Elementary school or less	21(8.8)	23(9.8)	0.774
Middle school	19(7.9)	16(6.8)	
High school	95(39.6)	84(35.9)	
College or more	105(43.8)	115(47.4)	
Monthly household income^b^			
<200	35(16.6)	24(11.8)	0.102
200 - <400	56(26.5)	72(35.3)	
>400	120(56.9)	108(52.9)	
Marital status			
Married	218(90.8)	215(90.0)	0.480
Unmarried	8(3.3)	5(2.1)	
Divorced/Widowed	14(5.8)	19(8.0)	
Smoking status			
Nonsmoker	147(64.8)	158(69.3)	0.215
Former smoker	33(14.5)	37(16.3)	
Current smoker	47(20.7)	33(14.5)	
Alcohol consumption			
Nondrinker	87(36.4)	100(41.5)	0.208
Former drinker	7(2.9)	12(5.0)	
Current drinker	145(60.7)	129(53.5)	
Age at menarche (years)^c^			
≤13	35(22.0)	31(20.5)	0.680
14	34(21.4)	34(22.5)	
15	37(23.3)	28(18.5)	
≥16	53(33.3)	58(38.4)	
Menopause (yes)^c^	83(47.7)	79(43.9)	0.472
Age at menopause (years)^c^			
<46	16(21.1)	12(17.7)	0.185
46 - <49	14(18.4)	8(11.8)	
49 - <52	14(18.4)	23(33.8)	
≥52	32(42.1)	25(36.8)	
Type of menopause^c^			
Natural	57(71.3)	51(64.6)	0.366
Surgery, Other	23(28.8)	28(35.4)	
Postmenopausal hormone use (ever)^c^	28(36.8)	20(30.8)	0.448
Parity (yes)^c^	157(96.3)	159(98.2)	0.315

^**a**^First-degree relative.

^b^Unit is 10,000 Korean won.

^c^Only in women.

Table 2 presents the association between the biomarkers of thyroid function/autoimmunity and thyroid cancer risk. When the markers were categorized by the tertile distributions of the control group, the highest tertile of FT4 (OR = 1.73, 95% CI = 1.11 - 2.69) showed an increased risk of thyroid cancer, while the middle tertile of TSH (OR = 1.77, 95% CI = 1.14 - 2.74) was associated with thyroid cancer risk. In addition, TPOAb levels greater than 30 IU/mL (OR = 8.47, 95% CI = 5.39 - 13.33 for 30 - < 60 IU/mL and OR = 4.48, 95% CI = 2.59 - 7.76 for ≥60 IU/mL) were strongly associated with risk of thyroid cancer when compared with those whose TPOAb levels were less than 30 IU/mL.

**Table 2:** **The association between the biomarkers of thyroid function/autoimmunity and thyroid cancer risk**

	Controls (n = 257)	Cases (n = 257)	Crude OR (95% CI)	Adjusted OR (95% CI) ^c^
TT3 (ng/mL)				
<1.2	88(34.2)	79(30.7)	1.0(ref)	1.0(ref)
1.2 - <1.4	104(40.5)	109(42.4)	1.17(0.78 - 1.75)	1.18(0.78 - 1.79)
≥1.4	65(25.3)	69(26.9)	1.18(0.75 - 1.86)	1.21(0.75 - 1.94)
FT4 (ng/dL)				
<1.25	85(33.1)	68(26.5)	1.0(ref)	1.0(ref)
1.25 - <1.39	85(33.1)	75(29.2)	1.10(0.71 - 1.72)	1.05(0.66 - 1.65)
≥1.39	87(33.9)	114(44.4)	1.64(1.07 - 2.57)^*^	1.73(1.11 - 2.69)^*^
TSH (μIU/mL)				
<1.36	84(33.1)	59(23.2)	1.0(ref)	1.0(ref)
1.36 - <2.5	86(33.9)	111(43.7)	1.81(1.18 - 2.79)^*^	1.77(1.14 - 2.74)^*^
≥2.5	84(33.1)	84(33.1)	1.40(0.90 - 2.19)	1.37(0.87 - 2.16)
Tg (ng/mL)^a^				
0 - <3.9	77(33.8)	67(29.7)	1.0(ref)	1.0(ref)
3.9 - <7	74(32.5)	64(28.3)	0.98(0.61 - 1.56)	0.97(0.60 - 1.55)
≥7	77(33.8)	95(42.0)	1.40(0.90 - 2.17)	1.40(0.89 - 2.19)
TgAb (IU/mL)^b^				
<20	212(82.5)	203(79.0)	1.0(ref)	1.0(ref)
20 - <60	17(6.6)	25(9.7)	1.54(0.81 - 2.93)	1.58(0.82 - 3.06)
≥60	28(10.9)	29(11.3)	1.08(0.62 - 1.88)	1.13(0.64 - 2.00)
TPOAb (IU/mL)^b^				
<30	192(74.1)	77(30.0)	1.0(ref)	1.0(ref)
30 - <60	38(14.8)	131(51.0)	8.60(5.49 - 13.45)^*^	8.47(5.39 - 13.33)^*^
≥60	27(10.5)	49(19.1)	4.53(2.64 - 7.76)^*^	4.48(2.59 - 7.76)^*^

*Abbreviations:* *CI*, Confidence interval; *OR*, Odds ratio; *TT3*, Total triiodothyronine; *FT4*, Free thyroxine; *TSH*, Thyroid-stimulating hormone; *Tg*, Thyroglobulin; *TgAb*, Anti-thyroglobulin antibody; *TPOAb*, Anti-thyroperoxidase antibody.

^a^Analyzed only for TgAb-negative subjects; ^b^The detection limits used were 20 IU/mL for TgAb and 30 IU/mL for TPOAb; ^c^Adjusted for age, sex, BMI, and smoking.

^*^ *P* <0.05.

The associations of some markers with thyroid cancer risk appeared to be different when the data were stratified by sex, BMI, smoking status, or the duration of follow-up (Table 3). The association between FT4 levels and thyroid cancer risk was only significant among women or those with a BMI <23 kg/m^2^. The elevated risk for the middle tertile of TSH was only significant among men, those with a BMI ≥23 kg/m^2^, or former/current smokers. The levels of TT3, FT4, and TSH were associated with thyroid cancer risk only when the duration of follow-up was shorter than 3 years. However, in all of the analyses, the presence of TPOAb strongly elevated the risk of thyroid cancer. Additionally, we examined whether other known risk factors showed different distributions according to the presence of TPOAb, but no differences were observed (Additional file 1: Table S1).

**Table 3:** **The association between thyroid function/autoimmunity biomarkers and thyroid cancer risk, stratified by sex, BMI, smoking status, and the duration of follow-up** ^**a**^

	Sex		BMI (kg/m ^2^ )		Smoking		Follow-up duration	
	Men	Women	<23	≥23	Non-smoker	Former/Current smoker	< 3 years	≥3 years
Controls/Cases	77/77	180/180	125/95	132/162	147/158	80/70	97/171	160/86
TT3 (ng/mL)								
<1.2	1.0(ref)	1.0(ref)	1.0(ref)	1.0(ref)	1.0(ref)	1.0(ref)	1.0(ref)	1.0(ref)
1.2 - <1.4	1.66(0.68 - 4.04)	1.08(0.67 - 1.75)	1.16(0.63 - 2.15)	1.18(0.67 - 2.08)	0.92(0.55 - 1.54)	2.07(0.85 - 5.02)	1.81(1.03 - 3.20)^*^	0.93(0.47 - 1.83)
≥1.4	1.56(0.61 - 4.00)	1.22(0.69 - 2.14)	0.93(0.43 - 2.00)	1.50(0.81 - 2.78)	0.80(0.43 - 1.51)	1.97(0.80 - 4.89)	2.51(1.21 - 5.22)^*^	1.10(0.53 - 2.25)
FT4 (ng/dL)								
<1.25	1.0(ref)	1.0(ref)	1.0(ref)	1.0(ref)	1.0(ref)	1.0(ref)	1.0(ref)	1.0(ref)
1.25 - <1.39	0.65(0.23 - 1.80)	1.19(0.71 - 2.00)	1.37(0.62 - 2.89)	0.91(0.51 - 1.64)	0.82(0.46 - 1.46)	1.05(0.42 - 2.66)	1.58(0.84 - 2.97)	0.73(0.36 - 1.46)
≥1.39	1.44(0.53 - 3.88)	1.76(1.06 - 2.92)^*^	2.52(1.28 - 4.96)^*^	1.43(0.78 - 2.60)	1.46(0.83 - 2.56)	1.85(0.77 - 4.41)	3.01(1.57 - 5.77)^*^	1.10(0.57 - 2.10)
TSH (μIU/mL)								
<1.36	1.0(ref)	1.0(ref)	1.0(ref)	1.0(ref)	1.0(ref)	1.0(ref)	1.0(ref)	1.0(ref)
1.36 - <2.5	2.31(1.06 - 5.02)^*^	1.58(0.92 - 2.72)	1.16(0.59 - 2.29)	2.31(1.30 - 4.13)^*^	1.71(0.95 - 3.09)	2.44(1.14 - 5.24)^*^	2.07(1.11 - 3.88)^*^	1.57(0.80 - 3.06)
≥2.5	1.97(0.76 - 5.08)	1.16(0.68 - 1.97)	0.96(0.49 - 1.89)	1.82(0.98 - 3.37)	1.23(0.68 - 2.22)	1.33(0.53 - 3.38)	1.36(0.72 - 2.59)	1.41(0.70 - 2.83)
Tg (ng/mL)^b^								
0 - <3.9	1.0(ref)	1.0(ref)	1.0(ref)	1.0(ref)	1.0(ref)	1.0(ref)	1.0(ref)	1.0(ref)
3.9 - <7	1.09(0.46 - 2.57)	0.93(0.52 - 1.66)	1.09(0.52 - 2.30)	0.95(0.52 - 1.77)	0.77(0.41 - 1.43)	1.16(0.48 - 2.79)	1.75(0.87 - 3.52)	0.65(0.32 - 1.33)
≥7	2.98(1.23 - 7.17)^*^	1.08(0.63 - 1.83)	1.09(0.55 - 2.14)	1.89(1.02 - 3.49)^*^	0.98(0.55 - 1.75)	2.52(1.05 - 6.03)^*^	1.80(0.96 - 3.36)	1.06(0.54 - 2.10)
TgAb (IU/mL)^c^								
<20	1.0(ref)	1.0(ref)	1.0(ref)	1.0(ref)	1.0(ref)	1.0(ref)	1.0(ref)	1.0(ref)
20 - <60	1.77(0.31 - 10.20)	1.56(0.77 - 3.19)	1.92(0.77 - 4.79)	1.27(0.50 - 3.26)	1.54(0.71 - 3.33)	1.33(0.28 - 6.26)	1.25(0.55 - 2.83)	1.17(0.35 - 3.89)
≥60	0.25(0.03 - 2.22)	1.31(0.71 - 2.41)	1.16(0.49 - 2.77)	1.11(0.52 - 2.37)	1.14(0.53 - 2.47)	1.07(0.30 - 3.79)	1.67(0.71 - 3.92)	0.77(0.32 - 1.87)
TPOAb (IU/mL)^c^								
<30	1.0(ref)	1.0(ref)	1.0(ref)	1.0(ref)	1.0(ref)	1.0(ref)	1.0(ref)	1.0(ref)
30 - <60	5.59(2.57 - 12.16)^*^	10.67(6.03 - 18.88)^*^	8.77(4.30 - 17.86)^*^	8.40(4.62 - 15.24)^*^	10.96(5.85 - 20.53)^*^	4.97(2.31 - 10.69)^*^	6.10(3.10 - 11.67)^*^	14.75(2.36 - 29.56)^*^
≥60	3.89(1.05 - 14.41)^*^	4.60(2.50 - 8.46)^*^	4.43(1.95 - 10.04)^*^	4.41(2.10 - 9.26)^*^	3.52(1.78 - 6.96)^*^	6.35(1.80 - 22.41)^*^	4.54(2.10 - 9.84)^*^	4.32(1.77 - 10.52)^*^

*Abbreviations:* *BMI*, Body mass index; *CI*, Confidence interval; *OR*, Odds ratio; *TT3*, Total triiodothyronine; *FT4*, Free thyroxine; *TSH*, Thyroid-stimulating hormone; Tg, thyroglobulin; *TgAb*, Anti-thyroglobulin antibody; *TPOAb*, Anti-thyroperoxidase antibody.

^a^Data were analyzed using multivariate logistic regression models which were adjusted for age, sex, BMI, and smoking; ^b^Analyzed only for TgAb-negative subjects; ^c^The detection limits used were 20 IU/mL for TgAb and 30 IU/mL for TPOAb.

^*^ *P* <0.05.

Finally, we examined the role of TPOAb in the association between the other biomarkers (TT3, FT4, TSH, Tg, and TgAb) and thyroid cancer risk (Table 4). The association between FT4 and thyroid cancer risk was stronger among those with TPOAb levels <30 IU/mL (OR = 2.12, 95% CI = 1.06 - 4.24).

**Table 4:** **The association between thyroid function/autoimmunity biomarkers and thyroid cancer risk, stratified by the presence of TPOAb**

	TPOAb (IU/mL)			
	<30		≥30	
	Controls/cases	Adjusted OR (95% CI) ^c^	Controls/cases	Adjusted OR (95% CI) ^c^
TT3 (ng/mL)				
<1.2	67/29	1.0(ref)	21/50	1.0(ref)
1.2 - <1.4	74/32	1.03(0.55 - 1.91)	30/77	1.05(0.53 - 2.07)
≥1.4	51/16	0.71(0.34 - 1.49)	14/53	1.71(0.76 - 3.85)
FT4 (ng/dL)				
<1.25	66/18	1.0(ref)	19/50	1.0(ref)
1.25 - <1.39	62/21	1.14(0.55 - 2.40)	23/54	0.87(0.42 - 1.80)
≥1.39	64/38	2.12(1.06 - 4.24)^*^	23/76	1.37(0.66 - 2.83)
TSH (μIU/mL)				
<1.36	66/16	1.0(ref)	18/43	1.0(ref)
1.36 - <2.5	63/32	2.00(1.00 - 4.01)^*^	23/79	1.48(0.73 - 3.00)
≥2.5	63/28	1.82(0.89 - 3.72)	21/56	1.14(0.54 - 2.40)
Tg (ng/mL)^a^				
0 - <3.9	58/22	1.0(ref)	19/45	1.0(ref)
3.9 - <7	59/18	0.70(0.34 - 1.44)	15/46	1.28(0.57 - 2.86)
≥7	63/32	1.21(0.63 - 2.32)	14/63	1.95(0.87 - 4.38)
TgAb (U/mL)^b^				
<20	170/69	1.0(ref)	42/134	1.0(ref)
≥20	22/8	0.95(0.40 - 2.26)	23/46	0.59(0.31 - 1.14)

*Abbreviations:* *CI*, Confidence interval; *OR*, Odds ratio; *TT3*, Total triiodothyronine; *FT4*, Free thyroxine; *TSH*, Thyroid-stimulating hormone; *Tg*, Thyroglobulin; *TgAb*, Anti-thyroglobulin antibody; *TPOAb*, Anti-thyroperoxidase antibody.

^a^Analyzed only for TgAb-negative subjects; ^b^The detection limits used were 20 IU/mL for TgAb, and we combined these groups into two groups because of the small sample sizes; ^c^Adjusted for age, sex, BMI, and smoking.

^*^ *P* <0.05.

## Discussion

This study prospectively investigated the association between biomarkers of thyroid function/autoimmunity and thyroid cancer risk and found that differences in the levels of thyroid biomarkers, particularly TPOAb, could predict the incidence of thyroid cancer.

Several studies have examined the association between thyroid function and thyroid cancer risk [6–8, 16, 17]. A large population-based cohort study from Taiwan [8] has investigated the incidence of cancer in patients with hyperthyroidism and found that patients with hyperthyroidism were at an increased risk for thyroid cancer. This group also reported that the duration of hyperthyroidism was related to increased risk of thyroid cancer. In the present study, the levels of thyroid hormones were normal in most of the study participants. However, relatively higher levels of FT4 showed a positive association with thyroid cancer risk. Because the association between thyroid hormones and thyroid cancer risk has not been sufficiently studied, the underlying mechanisms still remain unclear. Pellegriti *et al.* reported that circulating TSH receptor-stimulating antibodies (TSHR-Abs) were present in all patients with Graves’ disease [18], implying an association between TSHR-Abs and elevated levels of thyroid hormones. TSHR-Abs are known to stimulate the same intracellular signal pathways as TSH, which has mitogenic and antiapoptotic effects on thyroid follicular cells and thus may play a role in thyroid cancer initiation [19].

The positive association between TSH levels and thyroid cancer risk has been reported in some studies [6, 7, 16, 17], implying that high TSH levels may play a key role in the initiation of thyroid carcinogenesis. TSH has a proliferative effect on thyroid cell growth that is most likely mediated by TSH receptors on tumor cells [17]. However, some studies did not find an association between TSH levels and thyroid cancer risk [20]. Our study demonstrated that the highest tertile of TSH levels did not show any association with thyroid cancer, but the medium tertile of TSH levels seemed to slightly increase thyroid cancer risk.

Tg is produced by normal thyroid tissue and neoplastic follicular cells; therefore, serum Tg measurements can be used as specific and sensitive tumor markers of differentiated thyroid cancer in clinical practice [21]. The level of serum Tg is known to aid in the detection of residual, recurrent, or metastatic disease rather than in determining the incidence of thyroid cancer [22], but the role of Tg in the initiation of thyroid cancer remains unclear. However, this study has found that Tg is positively associated with thyroid cancer risk only among lean people, men, or smokers.

A high prevalence of thyroid cancer in those with autoimmune thyroid diseases [23–25] and systemic autoimmune diseases [26] may imply the possible association between thyroid autoimmunity and cancer risk. Kim *et al.*[24] found an elevated risk of papillary thyroid cancer in Korean patients with Hashimoto’s thyroiditis with elevated levels of TPOAb. Antonelli *et al*. [26] reported the higher prevalence of papillary thyroid cancer in systemic lupus erythematosus patients, particularly in patients with thyroid autoimmunity. The results of these studies suggest that the risk of thyroid cancer is strongly associated with elevated levels of TPOAb. TPO is a membrane protein that catalyzes thyroid hormone synthesis; thus, the presence of TPOAb in the blood may reflect an alteration in the immune system and lymphocytic infiltration in the thyroid [27]. TPOAb may destroy thyroid tissue as well as cytokines produced by infiltrating inflammatory cells, which may contribute to inflammation-induced carcinogenesis [25, 28, 29]. Furthermore, the presence of TPOAb could be associated with thyroid function. In a study using the NHANES III survey from the United States, Hollowell *et al.* reported an association between TPOAb and overt thyroid dysfunction [30]. We also observed that the presence of TPOAb may affect the association between FT4 levels and the risk of thyroid cancer.

Several factors may modify the association between thyroid abnormalities/thyroid autoimmunity and thyroid cancer risk. First, the effect of FT4 and TSH on thyroid cancer risk was affected by obesity status in the present study. In addition, previous studies have reported a positive association between obesity and thyroid cancer [4, 14]. It has also been proposed that obesity may affect the secretion of certain hormones such as insulin and sex steroids, which may act on the thyroid to stimulate cell proliferation and suppress apoptosis [31]. Second, this study also found that the levels of TSH and Tg were associated with thyroid cancer risk only among smokers. Smoking is known to have a negative association with the risk of thyroid cancer [13, 32], possibly by exerting anti-estrogenic effects or by affecting the immune system through nicotinic anti-inflammatory pathways [33–35]. Additionally, smoking is known to decrease the levels of TSH and the positivity of thyroid autoantibodies [36], which were reported to be positively associated with thyroid cancer risk. Third, the association of these biomarkers with thyroid cancer was different in men and women. The higher levels of TPOAb in women and the higher prevalence of smokers in men may partly explain the observed differences in the incidence of thyroid cancer based on sex [32]. A negative association between TPOAb and smoking was also reported [37].

The present study has strengths in its study design in terms of ascertaining thyroid cancer patients within a prospective cohort. This study design allowed for determining the potential role of pre-diagnostic serum levels of biomarkers on thyroid cancer risk. In addition, the controls were derived from the same cohort as the cases; thus, the potential selection bias that can occur with a conventional case–control study was minimized. However, the findings from the present study should be interpreted with caution because of several limitations. First, the duration of follow-up in this study was relatively short. However, the association between thyroid cancer and the levels of TPOAb was not modified by duration of follow-up in this study. Second, we lacked detailed information on the specifics of the thyroid cancer, e.g., tumor stage and histological type, because case ascertainment was performed by data linkage with the cancer registry; therefore, we could not include these variables in our analyses. Third, the sample size was relatively small, especially for a stratified analysis. Finally, the study population consisted of participants in a cancer-screening program; thus, these individuals may pay more attention to their health status and may not be representative of the general Korean population.

## Conclusions

We found that the levels of biomarkers of thyroid function and autoimmunity could provide additional information for predicting thyroid malignancy. Particularly, the presence of TPOAb seems to be a strong predictor of thyroid cancer. Interestingly, most participants who showed positive associations between these biomarkers and thyroid cancer risk were in the normal ranges of these markers and may not have had any symptoms of thyroid disease. Therefore, we cautiously suggest that careful monitoring of these biomarkers, even within the normal range, may be helpful for identifying those at high risk for thyroid cancer and for enhancing the likelihood of early detection in Koreans.

## Electronic supplementary material

Additional file 1: Table S1: General characteristics of the study subjects according to the presence of TPOAb. (PDF 46 KB)

## Abbreviations

BMI: Body mass index
CIs: Confidence intervals
CSCS: Cancer screenee cohort study
ELCLIA: Electrochemiluminescence immunoassay
FT4: Free thyroxine
KCCR: The Korea central cancer registry
ORs: Odds ratios
Tg: Thyroglobulin
TgAb: Anti-thyroglobulin
TPOAb: Anti-thyroperoxidase
TSH: Thyroid-stimulating hormone
TSHR-Abs: TSH receptor-stimulating antibodies
TT3: Triiodothyronine.

## References

[1] Pellegriti G, Frasca F, Regalbuto C, Squatrito S, Vigneri R (2013). Worldwide increasing incidence of thyroid cancer: update on epidemiology and risk factors. J Cancer Epidemiol.

[2] Jung KW, Won YJ, Kong HJ, Oh CM, Seo HG, Lee JS (2013). Prediction of cancer incidence and mortality in Korea, 2013. Cancer Res Treat.

[3] Verkooijen HM, Fioretta G, Pache JC, Franceschi S, Raymond L, Schubert H, Bouchardy C (2003). Diagnostic changes as a reason for the increase in papillary thyroid cancer incidence in Geneva, Switzerland. Cancer Causes Control.

[4] Peterson E, De P, Nuttall R (2012). BMI, diet and female reproductive factors as risks for thyroid cancer: a systematic review. PLoS One.

[5] Navarro Silvera SA, Miller AB, Rohan TE (2005). Risk factors for thyroid cancer: a prospective cohort study. Int J Cancer.

[6] Haymart MR, Repplinger DJ, Leverson GE, Elson DF, Sippel RS, Jaume JC, Chen H (2008). Higher serum thyroid stimulating hormone level in thyroid nodule patients is associated with greater risks of differentiated thyroid cancer and advanced tumor stage. J Clin Endocrinol Metab.

[7] Boelaert K, Horacek J, Holder RL, Watkinson JC, Sheppard MC, Franklyn JA (2006). Serum thyrotropin concentration as a novel predictor of malignancy in thyroid nodules investigated by fine-needle aspiration. J Clin Endocrinol Metab.

[8] Yeh NC, Chou CW, Weng SF, Yang CY, Yen FC, Lee SY, Wang JJ, Tien KJ (2013). Hyperthyroidism and thyroid cancer risk: a population-based cohort study. Exp Clin Endocrinol Diabetes.

[9] Kim ES, Lim DJ, Baek KH, Lee JM, Kim MK, Kwon HS, Song KH, Kang MI, Cha BY, Lee KW, Son HY (2010). Thyroglobulin antibody is associated with increased cancer risk in thyroid nodules. Thyroid.

[10] Moeller LC, Führer D (2013). Thyroid hormone, thyroid hormone receptors, and cancer: a clinical perspective. Endocr Relat Cancer.

[11] Sherman SI (2003). Thyroid carcinoma. Lancet.

[12] Kitahara CM, Linet MS, Beane Freeman LE, Check DP, Church TR, Park Y, Purdue MP, Schairer C, Berrington de González A (2012). Cigarette smoking, alcohol intake, and thyroid cancer risk: a pooled analysis of five prospective studies in the United States. Cancer Causes Control.

[13] Kitahara CM, Platz EA, Freeman LE, Hsing AW, Linet MS, Park Y, Schairer C, Schatzkin A, Shikany JM, Berrington de González A (2011). Obesity and thyroid cancer risk among U.S. men and women: a pooled analysis of five prospective studies. Cancer Epidemiol Biomarkers Prev.

[14] Agudo A, Sala N, Pera G, Capellá G, Berenguer A, García N, Palli D, Boeing H, Del Giudice G, Saieva C, Carneiro F, Berrino F, Sacerdote C, Tumino R, Panico S, Berglund G, Simán H, Stenling R, Hallmans G, Martínez C, Bilbao R, Barricarte A, Navarro C, Quirós JR, Allen N, Key T, Bingham S, Khaw KT, Linseisen J, Nagel G (2006). Polymorphisms in metabolic genes related to tobacco smoke and the risk of gastric cancer in the European prospective investigation into cancer and nutrition. Cancer Epidemiol Biomarkers Prev.

[15] Kim HK, Yoon JH, Kim SJ, Cho JS, Kweon SS, Kang HC (2013). Higher TSH level is a risk factor for differentiated thyroid cancer. Clin Endocrinol (Oxf).

[16] Fiore E, Vitti P (2012). Serum TSH and risk of papillary thyroid cancer in nodular thyroid disease. J Clin Endocrinol Metab.

[17] Pellegriti G, Mannarino C, Russo M, Terranova R, Marturano I, Vigneri R, Belfiore A (2013). Increased mortality in patients with differentiated thyroid cancer associated with Graves’ disease. J Clin Endocrinol Metab.

[18] Morshed SA, Latif R, Davies TF (2009). Characterization of thyrotropin receptor antibody-induced signaling cascades. Endocrinology.

[19] Hrafnkelsson J, Tulinius H, Kjeld M, Sigvaldason H, Jónasson JG (2000). Serum thyroglobulin as a risk factor for thyroid carcinoma. Acta Oncol.

[20] Shivaraj G, Prakash BD, Sonal V, Shruthi K, Vinayak H, Avinash M (2009). Thyroid function tests: a review. Eur Rev Med Pharmacol Sci.

[21] Park do J, Lim JA, Kim TH, Choi HS, Ahn HY, Lee EK, Kim KW, Park YJ, Yi KH, Cho BY (2012). Serum thyroglobulin level measured after thyroxine withdrawal is useful to predict further recurrence in whole body scan-negative papillary thyroid cancer patients after reoperation. Endocr J.

[22] Zhang L, Li H, Ji QH, Zhu YX, Wang ZY, Wang Y, Huang CP, Shen Q, Li DS, Wu Y (2012). The clinical features of papillary thyroid cancer in Hashimoto’s thyroiditis patients from an area with a high prevalence of Hashimoto’s disease. BMC Cancer.

[23] Kim KW, Park YJ, Kim EH, Park SY, Park DJ, Ahn SH, Park do J, Jang HC, Cho BY (2011). Elevated risk of papillary thyroid cancer in Korean patients with Hashimoto’s thyroiditis. Head Neck.

[24] Azizi G, Malchoff CD (2011). Autoimmune thyroid disease: a risk factor for thyroid cancer. Endocr Pract.

[25] Fiore E, Rago T, Scutari M, Ugolini C, Proietti A, Di Coscio G, Provenzale MA, Berti P, Grasso L, Mariotti S, Pinchera A, Vitti P (2009). Papillary thyroid cancer, although strongly associated with lymphocytic infiltration on histology, is only weakly predicted by serum thyroid auto-antibodies in patients with nodular thyroid diseases. J Endocrinol Invest.

[26] Antonelli A, Mosca M, Fallahi P, Neri R, Ferrari SM, D’Ascanio A, Ghiri E, Carli L, Miccoli P, Bombardieri S (2010). Thyroid cancer in systemic lupus erythematosus: a case–control study. J Clin Endocrinol Metab.

[27] Yoshida H, Amino N, Yagawa K, Uemura K, Satoh M, Miyai K, Kumahara Y (1978). Association of serum antithyroid antibodies with lymphocytic infiltration of the thyroid gland: studies of seventy autopsied cases. J Clin Endocrinol Metab.

[28] Feldt-Rasmussen U, Rasmussen AK (2010). Autoimmunity in differentiated thyroid cancer: significance and related clinical problems. Hormones.

[29] Balkwill F (2004). Cancer and the chemokine network. Nat Rev Cancer.

[30] Hollowell JG, Staehling NW, Flanders WD, Hannon WH, Gunter EW, Spencer CA, Braverman LE (2002). Serum TSH, T(4), and thyroid antibodies in the United States population (1988 to 1994): National Health and Nutrition Examination Survey (NHANES III). J Clin Endocrinol Metab.

[31] Roberts DL, Dive C, Renehan AG (2010). Biological mechanisms linking obesity and cancer risk: new perspectives. Annu Rev Med.

[32] Mack WJ, Preston-Martin S, Dal Maso L, Galanti R, Xiang M, Franceschi S, Hallquist A, Jin F, Kolonel L, La Vecchia C, Levi F, Linos A, Lund E, McTiernan A, Mabuchi K, Negri E, Wingren G, Ron E (2003). A pooled analysis of case–control studies of thyroid cancer: cigarette smoking and consumption of alcohol, coffee, and tea. Cancer Causes Control.

[33] Brand JS, Chan MF, Dowsett M, Folkerd E, Wareham NJ, Luben RN, van der Schouw YT, Khaw KT (2011). Cigarette smoking and endogenous sex hormones in postmenopausal women. J Clin Endocrinol Metab.

[34] Shiels MS, Rohrmann S, Menke A, Selvin E, Crespo CJ, Rifai N, Dobs A, Feinleib M, Guallar E, Platz EA (2009). Association of cigarette smoking, alcohol consumption, and physical activity with sex steroid hormone levels in US men. Cancer Causes Control.

[35] Scott DA, Martin M (2006). Exploitation of the nicotinic anti-inflammatory pathway for the treatment of epithelial inflammatory diseases. World J Gastroenterol.

[36] Belin RM, Astor BC, Powe NR, Ladenson PW (2004). Smoke exposure is associated with a lower prevalence of serum thyroid autoantibodies and thyrotropin concentration elevation and a higher prevalence of mild thyrotropin concentration suppression in the third National Health and Nutrition Examination Survey (NHANES III). J Clin Endocrinol Metab.

[37] Pedersen IB, Laurberg P, Knudsen N, Jørgensen T, Perrild H, Ovesen L, Rasmussen LB (2008). Smoking is negatively associated with the presence of thyroglobulin autoantibody and to a lesser degree with thyroid peroxidase autoantibody in serum: a population study. Eur J Endocrinol.

[CR38] The pre-publication history for this paper can be accessed here: http://www.biomedcentral.com/1471-2407/14/873/prepub

