# Supplementary material for: Biomarkers of thyroid function and autoimmunity for predicting high-risk groups of thyroid cancer: a nested case–control study
Source: BMC Cancer. 2014 Nov 24;14:873. doi: 10.1186/1471-2407-14-873 (PMC4289269; doi:10.1186/1471-2407-14-873)
Supplement: Supplementary file 1 — Additional file 1: Table S1: General characteristics of the study subjects according to the presence of TPOAb. (PDF 46 KB) [file 12885_2014_5123_MOESM1_ESM.pdf]

Supplemental Table 1. General characteristics of the study subjects according to the presence of TPOAb

	**TPOAb(IU/mL)**		
	**<30**	**≥30**	***P*-value**
Age (years), mean±SD	49.4±9.1	49.5±8.7	0.900
Gender			
Male	82(31.1)	71(29.1)	0.630
Female	182(68.9)	173(70.9)	
BMI (kg/m^2)			
<23	127(47.2)	93(38.0)	0.070
23−<25	73(27.1)	70(28.6)	
≥ 25	69(25.7)	82(33.5)	
Family history of cancer (yes)[a]	95(39.8)	100(46.1)	0.172
Smoking status			
Nonsmoker	158(65.6)	147(68.7)	0.775
Former smoker	39(16.2)	31(14.5)	
Current smoker	44(18.3)	36(16.8)	
Alcohol consumption			
Nondrinker	95(38.2)	83(35.9)	0.622
Former drinker	12(4.8)	8(3.5)	
Current drinker	142(57.0)	140(60.6)	
Age at menarche (years)[b]			
≤13	35(21.9)	31(20.7)	0.981
14	36(22.5)	32(21.3)	
15	33(20.6)	32(21.3)	
≥16	56(35.0)	55(36.7)	
Menopausal status[b]			
Pre-menopause	98(54.1)	94(54.3)	0.971
Post-menopause	83(45.9)	79(45.7)	
Age at menopause (years)[b]			
<46	15(19.2)	13(19.7)	0.953
46−<49	13(16.7)	9(13.6)	
49−<52	19(24.4)	18(27.3)	
≥52	31(39.7)	26(39.4)	
Type of menopause[b]			
Natural	60(74.1)	48(61.5)	0.091
Surgery, Other	21(25.9)	30(38.5)	
Postmenopausal hormone use (Ever)[b]	27(36.0)	21(31.8)	0.601
Parity (Yes)[b]	160(97.0)	156(97.5)	0.771

[a]First degree relative
[b]Analyzed only among women
